# Vapor-Assisted In Situ Synthesis of the Nb_2_CT*_x_*NS/NbO_2_F MXene Heterostructure for Enhanced Solar-Driven Photoelectrochemical Performance

**DOI:** 10.1021/acs.jpclett.4c02684

**Published:** 2024-12-27

**Authors:** Ying-Chih Pu, Yi-Chen Yu, Jen-An Shih, Yi-Li Chen, I-Wen Peter Chen

**Affiliations:** † Department of Chemistry, National Cheng Kung University, Tainan 701, Taiwan; ‡ Department of Materials Science, National University of Tainan, Tainan 70005, Taiwan; § eMemory Technology Inc. 8F, No. 5, Tai-Yuan 1st St., Jhubei City, Hsinchu County, 302082, Taiwan (https://www.ememory.com.tw/)

## Abstract

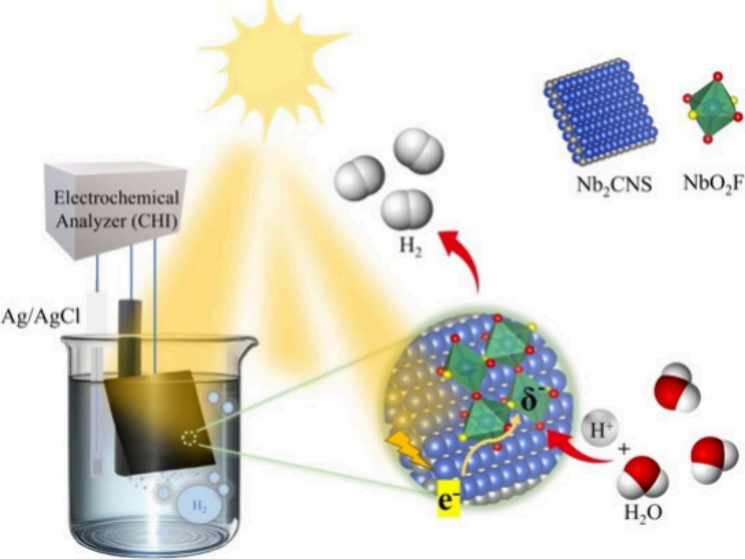

Photocatalytic water splitting holds great potential for transforming solar energy into valuable chemical products. However, obstacles such as the rapid recombination of electron–hole pairs and insufficiently active surface areas of photocatalysts remain significant challenges. In this study, we present the first demonstration that lithium bis(trifluoromethanesulfonyl)imide vapor successfully etches aluminum from Nb_2_AlC MAX phase powders while concurrently forming NbO_2_F anchors on Nb_2_CT*_x_* nanosheet (Nb_2_CT*_x_*NS) MXene, leading to the in situ formation of a Nb_2_CT*_x_*NS/NbO_2_F heterostructure composite. This novel material exhibits a remarkable photoelectrochemical performance, achieving a current density of 252 μA cm^–2^, which is 1000 and 10 times greater than those of Nb_2_AlC MAX and Nb_2_C nanosheet MXene, respectively. These findings shed light on innovative approaches for developing photocatalytic materials via vapor-assisted synthesis, offering a promising pathway for advancing material discovery in both photo- and energy-related fields.

A novel class of two-dimensional transition metal carbides and nitrides, commonly known as MXenes, possess superior electrical conductivity.^1^ These materials have redox transition metal atoms arranged in their layered configuration, making them a fast-growing family of two-dimensional (2D) materials.^2,3^ MXenes are prepared by removing A-layer elements in the layered MAX phases, where M represents an early transition metal (such as Ti, Nb, etc.), A represents group 13–16 elements (such as Al, Si, etc.), and X represents carbon or nitrogen. The general formula for MXenes can be represented as M*_n_*X_*n*–1_T_*x*_ (with *n* ranging from 2 to 4), where T_*x*_ indicates the surface termination functional group. The functional group is typically denoted as -F, -O, -OH, and/or -Cl on the 2D surface.^4^ Due to their distinctive 2D structure, adjustable surface properties (hydrophobic or hydrophilic), and exceptional electrical conductivity (>6000 S cm^–1^),^5^ MXenes show promise in a wide range of applications.^6,7^ Strong connections between MXenes and various materials can easily be integrated owing to the variety of functional groups on the surfaces of MXenes. The superior electrical conductivity of MXenes allows for boosting charge carrier transfer from the materials to the MXene, and an interfacial barrier will be formed at the interface of the materials and MXene, which will improve the separation efficiency of electrons and holes. In addition, charge accumulation in MXenes can cause a negative shift and tuning of the Fermi level and improvement of the photoelectrocatalytic efficiency.^8^ With these novel properties, MXenes are some of the most attractive photocatalysts and cocatalysts and hence have been studied immensely. Following the pioneering work on Ti_3_C_2_ MXene synthesis in 2011,^9^ MXenes became heavily dependent on the selective etching of A-layer elements in MAX phases through HF solutions or Lewis acid metal molten salts.^5^ The ongoing challenges of MXene synthesis are therefore (1) to find nonsolution synthesis routes and (2) to find a general approach of MAX phase precursors.

In recent years, with the rapid development of a modern technology-based society, the enormous demand for energy has become one of the most important issues affecting human life. Water splitting to generate H_2_ and O_2_ is a sustainable approach for generating clean energy with minimized risk to the global environment, and photoelectrochemical (PEC) technology is considered one of the most promising water splitting methods because it utilizes the unlimited energy source of solar light and does not emit greenhouse gases such as CO_2_.^10^ Therefore, many semiconductor-based photocatalysts have been synthesized for PEC hydrogen production, including metal oxides, metal dichalcogenides, carbon nitride, etc. Some of the most attractive photoelectrocatalysts are niobium-based materials, with excellent chemical resistance and photochemical stability, and they have been used for chemical degradation reactions.^11^ In addition, the photoelectrocatalytic activity of Nb-based materials can be enhanced by loading noble metal oxide nanoparticles as cocatalysts to boost electron–hole separation. However, the high cost and scarcity of noble metals limit their large-scale and practical application. In this work, we present the first report on a vapor-assisted route to etch the Al-layer element of Nb_2_AlC MAX for synthesizing Nb_2_CT_*x*_ nanosheets (Nb_2_CT_*x*_NS) and a NbO_2_F composite photoelectrocatalyst (Nb_2_CT_*x*_NS/NbO_2_F) using lithium bis(trifluoromethanesulfonyl)imide (LiTFSI). The effects of these structures on the photoelectrochemical properties of Nb_2_CT_*x*_NS/NbO_2_F were systematically investigated with respect to H_2_ evolution. We observed that the photocurrent of the Nb_2_CT_*x*_NS/NbO_2_F composite photoelectrocatalyst was enhanced by 1 order of magnitude compared to those of other MXene-based materials under identical experimental conditions.^12^ Furthermore, we demonstrated the role of Nb_2_CT_*x*_NS as an effective charge transportation substrate, indicating the considerable value of MXene materials as cocatalysts in photoelectrocatalysis. All synthesis materials and characterization details are provided in the Supporting Information.

Achieving vapor-assisted delamination of pristine bulk MAX phase material to MXenes was verified by X-ray diffraction (XRD), scanning electron microscopy (SEM), transmission electron microscopy (TEM), and high-resolution TEM energy dispersive X-ray spectroscopy (HRTEM-EDS) images. An effective delamination of the Nb_2_AlC MAX phase is achieved using LiTFSI-vapor-assisted etching. Figure S1 illustrates the impact of the reaction temperature on MAX phase etching for MXene formation through the LiTFSI vapor etching process. When the reaction temperature was increased above 370 °C, the XRD spectrum resulted in a new peak at 2θ of 8.55°, signifying MXene formation. When the reaction temperature exceeded 370 °C, the MXene signal remained consistent, indicating complete conversion of the MAX phase to MXene. In consideration of energy conservation, the etching temperature was subsequently held at 370 °C for further examination. Additionally, NbO_2_F was simultaneously and chemically bonded on Nb_2_CT_*x*_NS MXene to form the Nb_2_CT_*x*_NS/NbO_2_F composite during the Nb_2_AlC MAX phase etching process. According to the literature,^13^ the measured XRD characteristic peaks (Figure S1) match the literature assignment of NbO_2_F. To unveil the etching structure, SEM was conducted. Figure 1a shows the Nb_2_AlC MAX phase, which is a layered–stacked structure. As demonstrated in panels b and c of Figure 1, the delaminated Nb_2_CT_*x*_NS/NbO_2_F produced by LiTFSI vapor-assisted etching exhibits a sheet-like structure, indicating complete etching of Nb_2_AlC MAX and the formation of an open structure identified as Nb_2_CT_*x*_NS with NbO_2_F nanoparticles bonded to it. Figure S2 shows that the delaminated Nb_2_CT_*x*_NS of MXenes is nearly transparent, indicating the successful synthesis of a thin layer of Nb_2_CT_*x*_NS. Selected area electron diffraction (SAED) shows the Nb_2_CT_*x*_NS with a hexagonal structure as shown in the inset of Figure S2, indicating the delamination process does not damage the structure of Nb_2_CT_*x*_NS MXene. In addition, Figure 1d shows high-angle annular dark-field scanning transmission electron microscopy (HAADF-STEM) images of Nb_2_CT_*x*_NS/NbO_2_F. The Nb_2_CT_*x*_NS/NbO_2_F sheets detect Nb, F, O, and C signals on the surface, and the detected Al signal was background noise. Hence, the LiTFSI vapor-assisted etching process emerges as a novel and effective method for synthesizing a Nb_2_CT_*x*_NS/NbO_2_F composite in a one-step synthesis. To compare the delamination performance, we utilized the CuCl_2_ molten salt etching method to prepare Nb_2_C MXene nanosheets, which was reported in the literature, as a reference. Figure S3 shows the molten salt prepared Nb_2_CNS accordion structure that is identical to that described in the literature.^5^ With regard to the molten salt method, we found the open structure of Nb_2_CNS was not significant and no nanocrystals were formed. Hence, according to the results mentioned above, the proposed vapor-assisted etching process offers a new and high-efficiency route for the synthesis of highly open structures of the MXene composite.^14^

**Figure 1:**
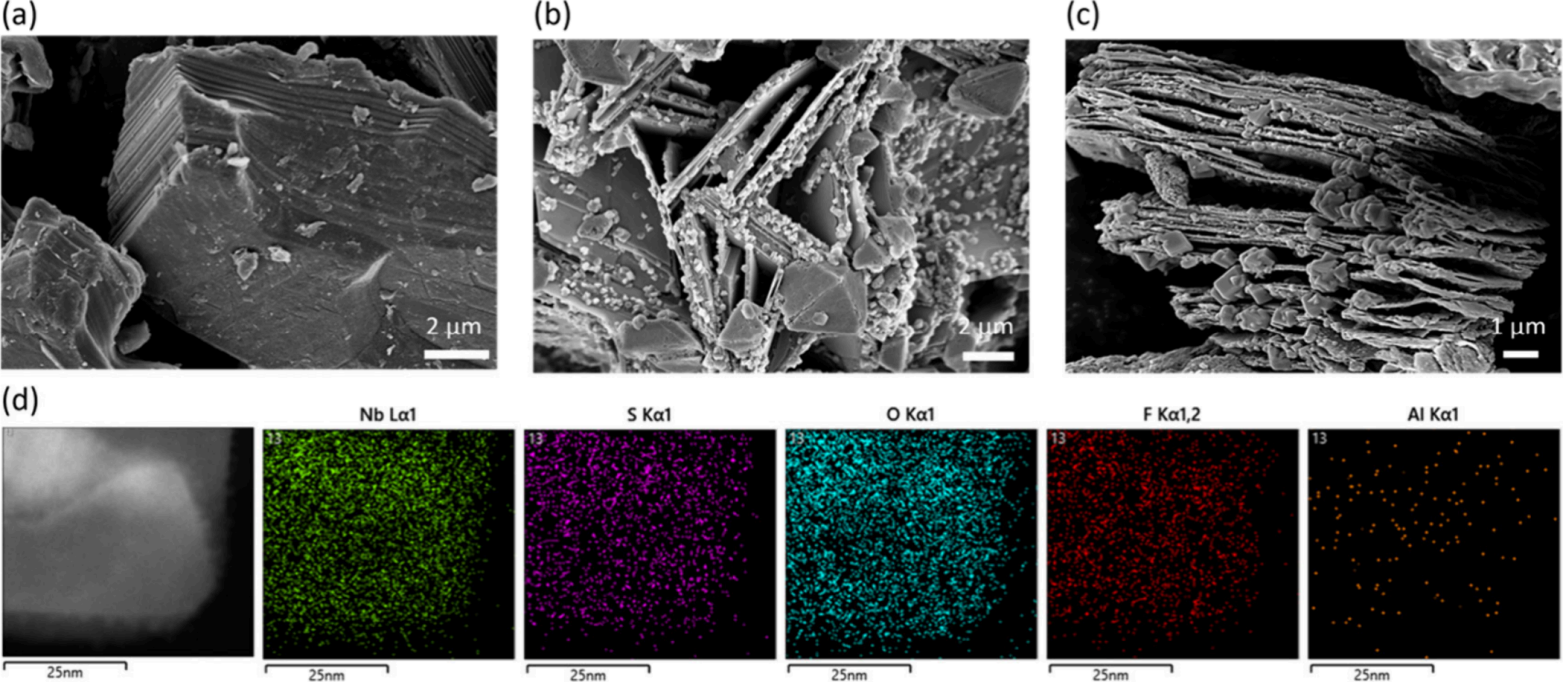
Morphological characterization of Mxenes: (a) Nb_2_AlC MAX and (b and c) Nb_2_CT_*x*_NS/NbO_2_F composite. (d) STEM-HAADF graph and EDS of the Nb_2_CT_*x*_NS/NbO_2_F composite.

The crystallographic phases of all of the synthesized MXenes were investigated by XRD. Figure 2a shows the characteristic diffraction peaks of Nb_2_AlC MAX, Nb_2_CNS MXene, and Nb_2_CT_*x*_NS/NbO_2_F MXene. The characteristic Al peak of the MXenes in the XRD spectra had disappeared, which means that CuCl_2_ and LiTFSI were effective HF-free etchants. Figure 2b shows values of 8.62° and 10.05°, respectively, indicating the distance of the *c*-axis of MAX that was successfully etched and the Nb_2_CT_*x*_ MXene layers of Nb_2_CT_*x*_NS/NbO_2_F were much larger than those of Nb_2_CNS MXene.^15,16^ Moreover, the full width at half-maximum (fwhm) of Nb_2_CT_*x*_NS/NbO_2_F is 8.62°, which is significantly smaller than the fwhm of 10.05° of Nb_2_CNS MXene, demonstrating the larger sheet size of Nb_2_CT_*x*_NS/NbO_2_F formation. Moreover, NbO_2_F nanocrystal peaks appeared in the Nb_2_CT_*x*_NS MXene, indicating the formation of the Nb_2_CT_*x*_NS/NbO_2_F composite via the one-step vapor-assisted synthesis method.^5^ This is the first time NbO_2_F chemical bonding has been performed on Nb_2_CT_*x*_NS via a facile synthesis route. Figure 2c shows the MXene peaks after the etching process, indicating that the planar structure remains in its original configuration.^17^ The Brunauer–Emmett–Teller (BET) method was employed to analyze the surface area, and the results are illustrated with nitrogen absorption–desorption isotherms in Figure 2d. The surface areas for Nb_2_AlC MAX, Nb_2_CNS, and Nb_2_CT_*x*_NS/NbO_2_F are 0.8756, 16.4351, and 22.6052 m^2^ g^–1^, respectively, indicating that vaporized LiTFSI is a suitable etchant for synthesizing the Nb_2_CT_*x*_NS/NbO_2_F composite. Moreover, the Barrett–Joyner–Halenda (BJH) pore size distribution was determined for Nb_2_AlC MAX, Nb_2_CNS, and Nb_2_CT_*x*_NS/NbO_2_F, as depicted in Figure 2e. The BJH average pore widths of Nb_2_CT_*x*_NS/NbO_2_F are centered at 14.98 and 61.17 nm, indicating the formation of a mixed pore distribution in the Nb_2_CT_*x*_NS/NbO_2_F MXene composite.

**Figure 2:**
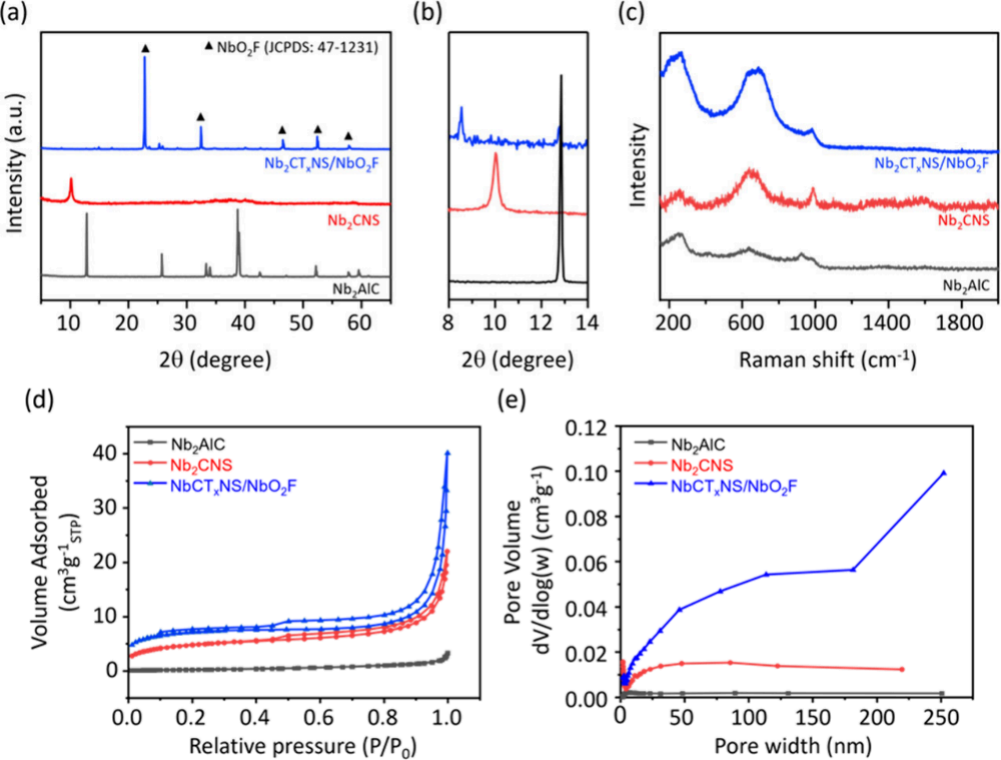
(a) XRD patterns, (b) magnified XRD patterns, and (c) Raman spectra of Nb_2_AlC MAX powder (bottom), Nb_2_CNS synthesized with the assistance of CuCl_2_ (middle), and Nb_2_CT_*x*_NS/NbO_2_F (top) synthesized with the LiTFSI vapor-assisted method. (d) Surface areas and (e) pore size distributions of the Nb_2_AlC MAX, Nb_2_CNS, and Nb_2_CT_*x*_NS/NbO_2_F MXene samples.

The band gaps of Nb_2_AlC MAX and Nb_2_CT_*x*_NS/NbO_2_F were measured at 1.87 and 2.45 eV, respectively.^18^ Notably, the literature suggests that the band gap of NbO_2_F stands at 3.35 eV.^5^ Consequently, the Nb_2_CT_*x*_NS/NbO_2_F composite effectively reduces the band gap of pure NbO_2_F; thus, vapor-assisted one-step synthesis of Nb_2_CT_*x*_NS/NbO_2_F could further enhance the photocatalytic performance. Photoluminescence (PL) spectroscopy measurement was carried out. PL emissions originate from the radiative recombination of photogenerated electron–hole pairs. Therefore, it is a useful method for understanding the recombination of electron–hole pairs in semiconductors by measuring their PL emission intensity. The PL emission spectra of Nb_2_AlC MAX, Nb_2_CT_*x*_NS/NbO_2_F, and Nb_2_CNS with 325 nm excitation at room temperature are displayed in Figure 3c. The emission band intensity of the Nb_2_CT_*x*_NS/NbO_2_F sample is observed, which suggests that Nb_2_CT_*x*_NS/NbO_2_F has a semiconductor property. Peaks in the PL emission spectrum of Nb_2_CT_*x*_NS/NbO_2_F appear at 475 and 530 nm, indicating NbO_2_F appearing in the Nb_2_CT_*x*_NS/NbO_2_F material.

**Figure 3:**
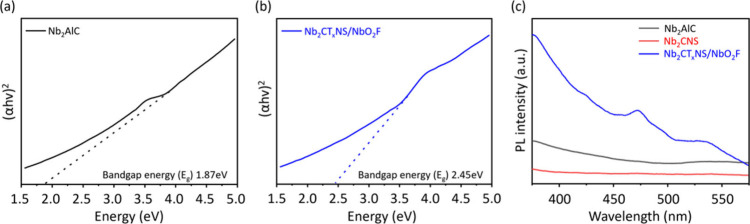
Plots of squared absorption coefficient (α*h*ν)^2^ vs energy *h*ν of (a) Nb_2_AlC MAX and (b) Nb_2_CT_*x*_NS/NbO_2_F. (c) PL spectra of Nb_2_AlC MAX, Nb_2_CNS, and Nb_2_CT_*x*_NS/NbO_2_F.

To understand the valence states, the interfacial interactions of each element in the Nb_2_CT_*x*_NS/NbO_2_F composite were further characterized by X-ray photoelectron spectroscopy (XPS) analysis. The high-resolution Nb 3d XPS spectra of Nb_2_C MXene (Figure 4a) displayed three peaks at 203.80 eV (Nb–C 3d_5/2_), 207.36 eV (NbC_*x*_O_*y*_ 3d_3/2_), and 210.11 eV [Nb(V) 3d_3/2_].^15,19^ The high-resolution C 1s XPS spectrum could be fitted into three peaks at 284.49 eV (C–C), 286.04 eV (C–H), and 288.65 eV (O–C=O) as shown in Figure 4b.^20^ In the O 1s XPS spectra (Figure 4c), peaks are detected at 530.33 eV (Nb–O–Nb) and 531.77 eV [Nb–(OH)].^19^Figure 4d shows the formation of Nb–F bonding in the Nb_2_CT_*x*_NS/NbO_2_F sample, indicating that the F from LiTFSI can be broken and attached to Nb during hydrothermal treatment. The limited S element of LiTFSI was also functionalized onto the surface of the Nb_2_CT_*x*_NS/NbO_2_F sample. Hence, XPS data echo the TEM and XRD characterization results, indicating the successful formation of Nb_2_CT_*x*_NS/NbO_2_F via the vapor-assisted etching method.

**Figure 4:**
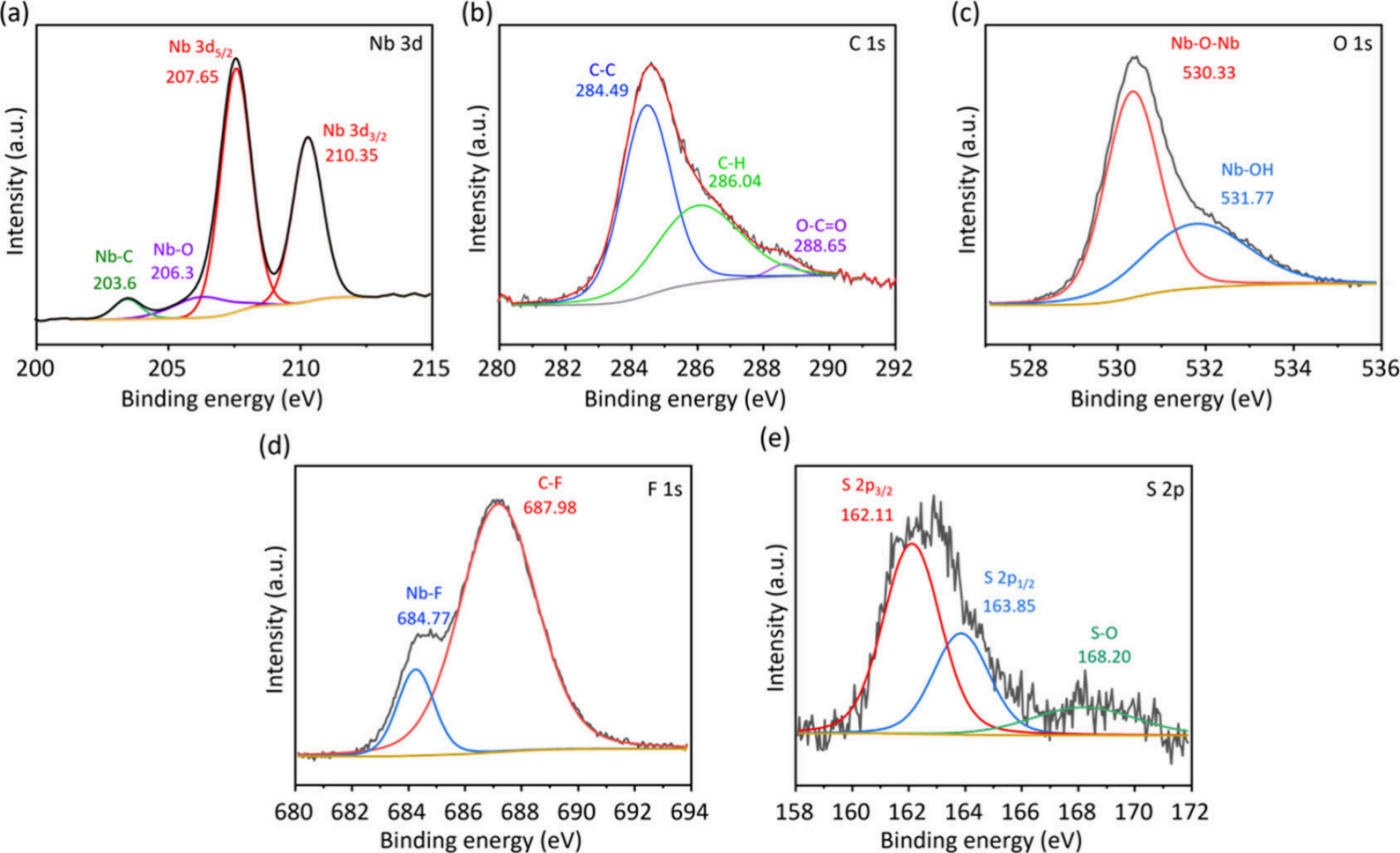
Representative deconvoluted XPS spectra of Nb_2_CT_*x*_NS/NbO_2_F: (a) Nb 3d, (b) C 2p, (c) O 1s, (d) F 1s, and (e) S 2p.

The PEC activity for the hydrogen evolution reaction (HER) of the MXenes was studied. The PEC properties were assessed in a complete PEC cell using an aqueous electrolyte under neutral and acidic conditions. Na_2_SO_4_ was utilized as the electrolyte for the neutral condition because it does not provide an environment for H^+^ generation. Hence, the intrinsic properties of the water splitting performance of the PEC catalysts can be clearly determined. Figure 5a shows the PEC performance of Nb_2_CNS and Nb_2_CT_*x*_NS/NbO_2_F at an applied potential of 0 V under the Na_2_SO_4_ condition. During the simulated sun irradiation, the changes in the PEC current density of Nb_2_AlC MAX, Nb_2_CNS, and Nb_2_CT_*x*_NS/NbO_2_F are 0, 2, and 50 μA cm^–2^, respectively. This means that Nb_2_CT_*x*_NS/NbO_2_F MXene shows a significant enhancement of photocurrent generation. Moreover, upon comparison with the previous results for oxidized Nb_2_CT_*x*_ determined via CO_2_ oxidation of Nb_2_CT_*x*_NS powder,^11^ Nb_2_CT_*x*_NS/NbO_2_F shows an enhancement of 1 order of magnitude. Lange et al. mentioned that upon irradiation with light under a nearly zero applied bias condition, pristine NbO_2_F shows zero photocurrent, indicating electron–hole recombination is faster than electron–hole transportation.^21^ Hence, we demonstrate that Nb_2_CT_*x*_NS in Nb_2_CT_*x*_NS/NbO_2_F provides a pathway to reduce the level of electron–hole recombination. We further measured Nb_2_CT_*x*_NS/NbO_2_F under harsh acidic conditions to discuss the photocurrent generation efficiency and the stability of the photocatalyst. The generated photocurrents of Nb_2_AlC MAX, Nb_2_CNS, and Nb_2_CT_*x*_NS/NbO_2_F are 0.2, 30, and 252 μA cm^–2^, respectively. After 35 light on/off cycles, Nb_2_CT_*x*_NS/NbO_2_F shows stable photocurrent generation, indicating Nb_2_CT_*x*_NS/NbO_2_F does not lose its intrinsic properties under the acidic condition. Compared to the literature, most of the Nb_2_C-based systems can generate an only limited photocurrent density signal for PEC application. For example, Su et al. showed the Nb_2_O_5_/C/Nb_2_C composite can generate a maximum photocurrent density of only 4.2 μA cm^–2^ under ultraviolet (UV) illumination and no photocurrent response under visible light illumination.^11^ Gao et al. showed the prepared Nb_2_C MXenes are sensitive to UV light, while the appearance of a photocurrent density signal of only 10 nA cm^–2^ is observed in the visible light region.^22^ Tayyab et al. synthesized a ternary In_2_S_3_/Nb_2_O_5_/Nb_2_C heterojunction for photocatalytic hydrogen production. The maximum peak photocurrent density of In_2_S_3_/Nb_2_O_5_/Nb_2_C is 4 μA cm^–2^ in the visible light region.^23^ To assess the photocurrent generation of the photocatalyst, electrochemical impedance spectroscopy (EIS) was conducted to elucidate the charge-transfer resistance. Specifically, a smaller radius of the arc in the EIS spectra indicates lower electron-transfer resistance on the surface of the photocatalyst electrodes, which generally suggests a more effective depletion of electron–hole pairs and enhanced interfacial charge transfer. As shown in Figure S4, the arc radius of the EIS Nyquist plots for Nb_2_CT*_x_*NS/Nb_2_O_5_F is noticeably smaller than that of Nb_2_CNS. The EIS Nyquist plots can be modeled using the equivalent electrical circuit depicted in the inset of Figure S4, where *R*_t_, *R*_s_, and CPE represent the interfacial charge-transfer resistance, electrolyte solution resistance, and constant phase element, respectively. The arc radius and *R*_t_ for Nb_2_CT*_x_*NS/Nb_2_O_5_F are the smallest among the samples, indicating the lowest resistance for interfacial charge transfer from the electrode to the electrolyte molecules. To further elucidate the performance of the Nb_2_CT*_x_*NS/Nb_2_O_5_F photoelectrode, linear sweep voltammetry (LSV) tests were conducted. As shown in Figure S5a, the LSV curve for the Nb_2_CT*_x_*NS/Nb_2_O_5_F photoelectrode exhibits a significant photocurrent enhancement of ∼250 μA cm^2^ upon simulated light on/off irradiation at an applied bias potential of −0.1 V (vs RHE). Additionally, Figure S5b demonstrates that as the applied bias potential becomes more negative both the light-induced photocurrent and the dark current increase accordingly. Hence, Nb_2_CT_*x*_NS is pivotal in Nb_2_CT_*x*_NS/NbO_2_F avoiding electron–hole recombination.

**Figure 5:**
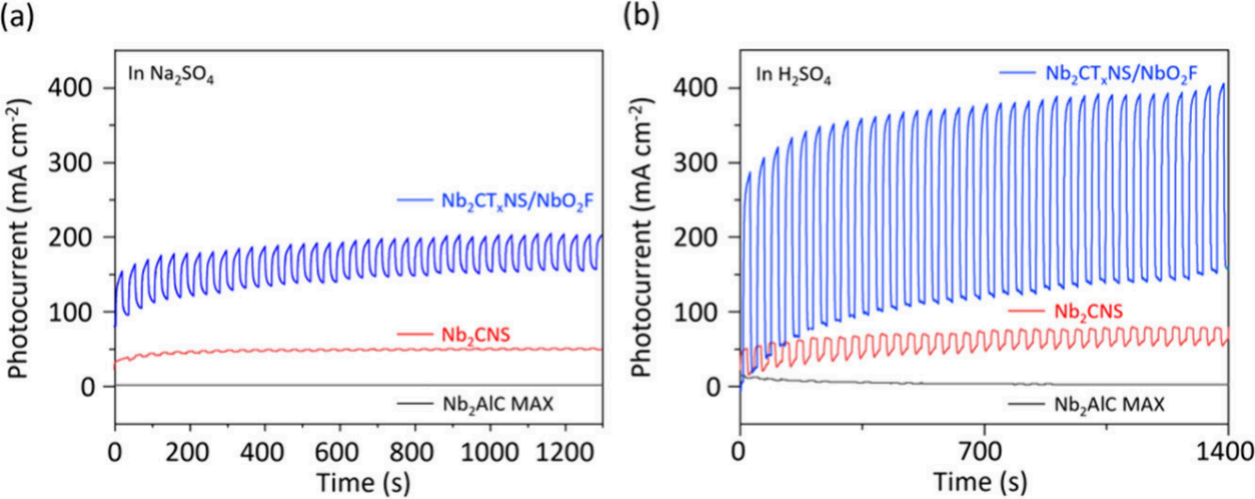
Visible light illumination testing of photocurrents of (a) Nb_2_AlC MAX, Nb_2_NCS, and Nb_2_CT_*x*_NS/NbO_2_F at an applied potential of 0 V under a 0.2 M Na_2_SO_4_ electrolyte and (b) Nb_2_AlC MAX, Nb_2_CT_*x*_NS/NbO_2_F, and Nb_2_CNS at an applied potential of 0 V under a 0.25 M H_2_SO_4_ electrolyte.

Photocatalytic hydrogen evolution under visible light irradiation was investigated by using a Nb_2_CT_*x*_NS/NbO_2_F composite photocatalyst. Figure 6a depicts the gas chromatography chromatogram of H_2_ evolution of Nb_2_CT_*x*_NS/NbO_2_F under a xenon lamp with a UV cutoff filter. Figure 6b shows that Nb_2_CT_*x*_NS exhibited only 1 μmol of H_2_ g^–1^ after 4 h due to rapid recombination of photogenerated charge carriers. In contrast, Nb_2_CT_*x*_NS/NbO_2_F shows significantly higher photocatalytic efficiency, with H_2_ evolution efficiencies of 1.5 and 0.25 μmol g^–1^ h^–1^ for Nb_2_CT_*x*_NS/NbO_2_F and Nb_2_CNS, respectively, under a neutral electrolyte. These findings demonstrate the crucial role of efficient charge carrier transportation in enhancing hydrogen generation, consistent with chronoamperometric measurements (Figure 5). This study marks the pioneering development of MXene-based composite photoelectrodes via vapor-assisted synthesis, suggesting promising future advancements in this field.

**Figure 6:**
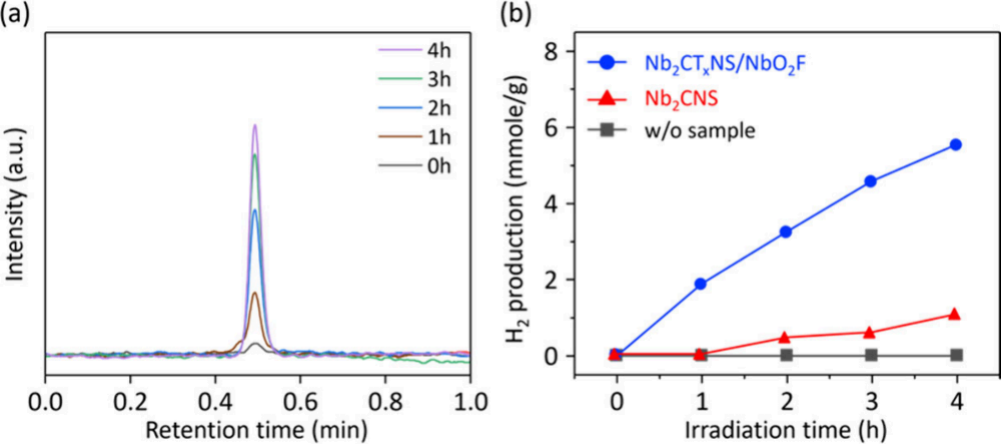
Product analysis of the Nb_2_CT_*x*_NS/NbO_2_F composite photocatalyst. (a) H_2_ evolution chromatogram monitored by gas chromatography during continuous working electrode operation under a xenon lamp with a UV cutoff filter in a 0.1 M Na_2_SO_4_ electrolyte solution. (b) Amounts of hydrogen evolved vs illumination time.

In this study, for the first time, we proposed a simple in situ one-step vapor etching approach for preparing the Nb_2_CT_*x*_NS/NbO_2_F composite photocatalyst directly. Belonging to the Nb-based systems, as an active material in working electrodes, the Nb_2_CT_*x*_NS/NbO_2_F composite photocatalyst shows the highest photocurrent density (252 μA cm^–2^ at 0 V) and outstanding stability under visible light illumination. Such a great enhancement in photocatalytic H_2_ evolution was due to the in situ synthesized NbO_2_F bonding on the Nb_2_C MXene surface to form superior interfacial contacts. The vapor-assisted synthesis method is simpler than the conventional photodeposition technique. It not only streamlines the process with fewer steps but also facilitates the formation of a well-developed semiconductor/MXene heterojunction. This heterojunction structure effectively boosts charge separation during the transfer of photogenerated carriers, resulting in enhanced photocatalytic HER performance. This understanding of the reactivity of MXenes opens up a versatile new avenue for the facile synthesis of MXene-based photocatalysts.

## Supporting Information Available

Effect of temperature of LiTFSI etching Al of Nb_2_AlC MAX powder, TEM graph and SAED of Nb_2_CT_*x*_NS/NbO_2_F, morphological characterization of Nb_2_CNS MXene treated with CuCl_2_, Nyquist plots of Nb_2_CT_*x*_NS/NbO_2_F and Nb_2_CNS, and LSV plots of the forward Nb_2_CT_*x*_NS/NbO_2_F photoelectrode (PDF)
