# Supplementary material for: Vapor-Assisted In Situ Synthesis of the Nb2CTxNS/NbO2F MXene Heterostructure for Enhanced Solar-Driven Photoelectrochemical Performance
Source: J Phys Chem Lett. 2024 Dec 27;16(1):333–8. doi: 10.1021/acs.jpclett.4c02684 (PMC11726660; doi:10.1021/acs.jpclett.4c02684)
Supplement: Supplementary file 1 — jz4c02684_si_001.pdf [file jz4c02684_si_001.pdf]

Supporting information

Vapor-Assisted In-Situ Synthesis of Nb_2CT_xNS/NbO_2F MXene Heterostructure for Enhanced Solar-Driven Photoelectrochemical Performance

Ying-Chih Pu, Yi-Chen Yu, Jen-An Shih, Yi-Li Chen, I-Wen Peter Chen*

Experimental section

Chemicals

Niobium aluminum carbide (Nb_2AlC; 98%) was purchased from 11 Technology Co.,Ltd. Lithium bis(trifluoromethanesulfonyl)imide (LiTFSI; >98%) was purchased from Tokyo Chemical Industry Co., Ltd. Hydrochloric acid (HCl; ≥ 37%) was purchased from Honeywell Fluka. Ammonium fluoride (NH_4F; 97%) was purchased from Showa Chemical Industry Co., Ltd. 1-Methyl-2-Pyrrolidinone (C_5H_9NO; 99%) was purchased from Echo Chemical Co., Ltd. Polyvinylidene difluoride (PVDF) and Super P were purchased from Acros and Timcal, respectively. $CuCl_2$, NaCl and KCl were purchase from Sigma-Aldrich. Ammonium peroxodisufate $(NH_4)_2S_2O_8$ (APS; 98%) purchased from Showa Chemical Industry Co., Ltd. All chemicals were used as received.

Synthesis of Nb_2CT_xNS /NbO_2F

For synthesis of Nb_2CT_xNS/NbO_2F, 0.3 g of Nb_2AlC MAX powder was mixed homogeneously with 1.2 g LiTFSI, and the mixed powder was involved in autoclave reactor at 330°C/370°C/400°C/420°C/450°C for 5 h. After reaction, the synthesized Nb_2CT_xNS/NbO_2F powder was thoroughly washed with 12 N HCl (37%) and 2.96 g NH_4F mixed solution to remove impurities. Then, we use DI water to wash the Nb_2CT_xNS/NbO_2F powder three times. The Nb_2CT_xNS/NbO_2F composite photoelectrocatalyst was freeze-dried and storage in ambient condition.

Synthesis of Nb_2C nanosheets

According to the literature, for synthesis of Nb_2C nanosheets (denoted as Nb_2CNS), 1g Nb_2AlC MAX powder mixed with 1.8 g $CuCl_2$, 0.52 g NaCl, and 0.66 g KCl and ground well for 5 min. Then, the mixture was placed in tube furnace and heated to 750°C at a heating rate of 4°C/min under argon filled condition for 5 h. The resulting products were thoroughly washed with deionized water to remove salts. Next, APS was used to eliminate any residual copper, followed by another wash with deionized water to remove any remaining APS. The final powders were collected by freeze-drying. Then the Nb_2CNS powder was storage in ambient condition.

Photoelectrochemical measurement for MXenes

A 500 W xenon lamp equipped with an AM 1.5 filter (providing an irradiance of 100 mW/cm^2) was utilized as the simulated sunlight source. A total of 6 mg of the photocatalysts were dispersed in 15 mL of a methanol and deionized water solution with a 1:4 volume ratio in the gas-tight reactor. During light exposure, gas samples

were periodically extracted from the reaction chamber by a gas tight syringe and introduced into a gas chromatograph (Shimadzu GC-2010, BID 2010 Plus) equipped with a barrier discharge ionization detector. A six-port valve was used to control the injection volume, maintaining a fixed sample size of 1 mL for each analysis.

Characterization

The structural properties of the prepared materials were characterized by fiber-coupled Raman spectrometer (Laser wavelength: 532 nm), X-ray diffraction (XRD; D8 Advance Eco, Bruker), X-ray photoelectron spectroscopy (XPS; Thermo), JEOL JEM-2100F CS STEM high-resolution transmission electron microscopy (HR-TEM; Hitachi, Tokyo, Japan), and scanning electron microscopy (SEM, ZEISS AURIGA) and energy-dispersive X-ray spectroscopy (EDS, ZEISS AURIGA). The surface area and pore size of the prepared MXene was obtained by using the Brunauer-Emmett-Teller (BET; ASAP2060; Micromeritics) method. The photoelectrochemical properties were tested using the electrochemical workstation CHI 7927E (CH Instruments Inc., Austin, TX, USA) with a 250 W Xe lamp (with 420 nm UV filter). In this three-electrode system, the materials, Ag/AgCl, and graphite act as a working electrode, reference electrode, and counter electrode, respectively.

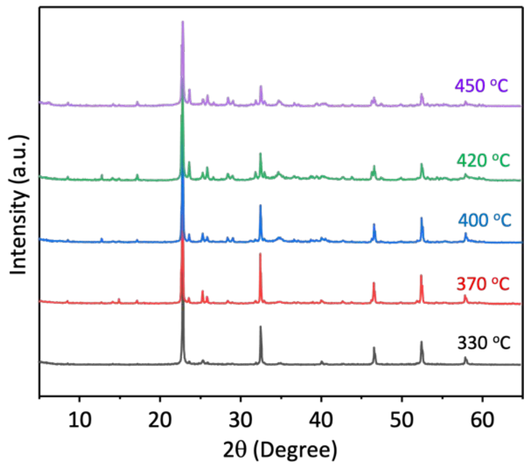

Figure S1. Effect of temperature of LiTFSI etching Al of Nb_2AlC MAX powder.

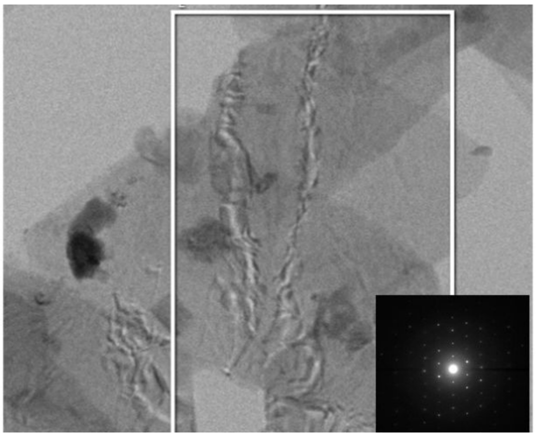

Figure S2. TEM graph and SAED.

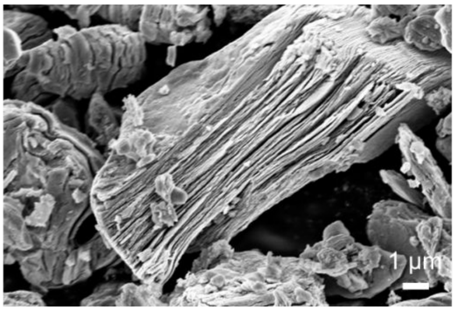

Figure S3. Morphological characterization of Nb_2CNS MXene treated via $CuCl_2$.

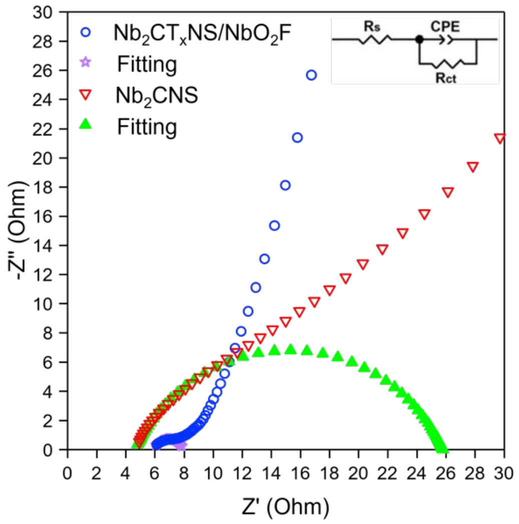

Figure S4. Nyquist plots of Nb_2CT_xNS/NbO_2F and Nb_2CNS.

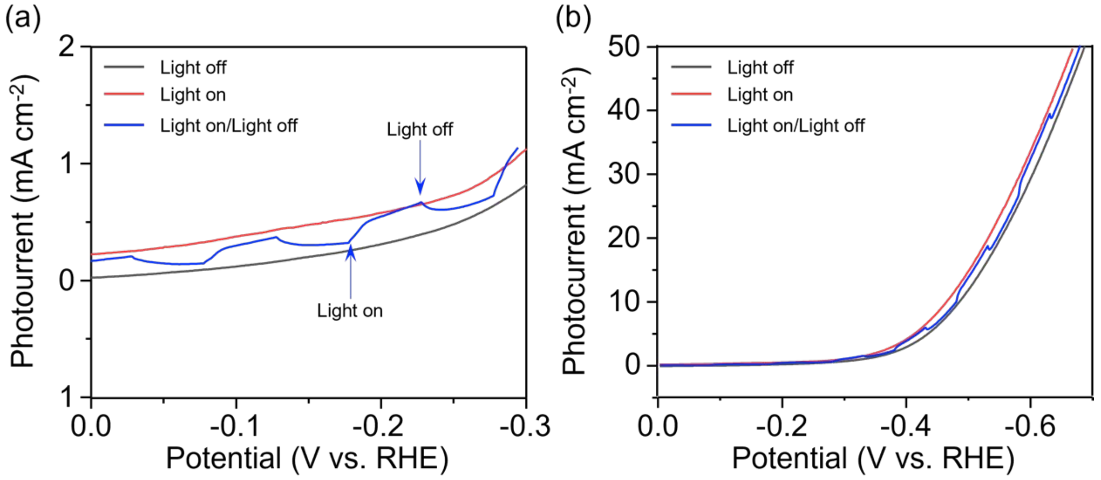

Figure S5. LSVs of forward Nb_2CT_xNS/NbO_2F photoelectrode. The scan rate is 1 mV/s.
